# Differential Inhibitor Sensitivity between Human Kinases VRK1 and VRK2

**DOI:** 10.1371/journal.pone.0023235

**Published:** 2011-08-04

**Authors:** Marta Vázquez-Cedeira, Iria Barcia-Sanjurjo, Marta Sanz-García, Ramiro Barcia, Pedro A. Lazo

**Affiliations:** 1 Experimental Therapeutics and Translational Oncology Program, Instituto de Biología Molecular y Celular del Cáncer, CSIC-Universidad de Salamanca, Salamanca, Spain; 2 Departamento de Bioquímica y Biología Molecular, Facultad de Veterinaria, Universidad de Santiago de Compostela, Lugo, Spain; Griffith University, Australia

## Abstract

Human vaccinia-related kinases (VRK1 and VRK2) are atypical active Ser-Thr kinases implicated in control of cell cycle entry, apoptosis and autophagy, and affect signalling by mitogen activated protein kinases (MAPK). The specific structural differences in VRK catalytic sites make them suitable candidates for development of specific inhibitors. In this work we have determined the sensitivity of VRK1 and VRK2 to kinase inhibitors, currently used in biological assays or in preclinical studies, in order to discriminate between the two proteins as well as with respect to the vaccinia virus B1R kinase. Both VRK proteins and vaccinia B1R are poorly inhibited by inhibitors of different types targeting Src, MEK1, B-Raf, JNK, p38, CK1, ATM, CHK1/2 and DNA-PK, and most of them have no effect even at 100 µM. Despite their low sensitivity, some of these inhibitors in the low micromolar range are able to discriminate between VRK1, VRK2 and B1R. VRK1 is more sensitive to staurosporine, RO-31-8220 and TDZD8. VRK2 is more sensitive to roscovitine, RO 31–8220, Cdk1 inhibitor, AZD7762, and IC261. Vaccinia virus B1R is more sensitive to staurosporine, KU55933, and RO 31–8220, but not to IC261. Thus, the three kinases present a different pattern of sensitivity to kinase inhibitors. This differential response to known inhibitors can provide a structural framework for VRK1 or VRK2 specific inhibitors with low or no cross-inhibition. The development of highly specific VRK1 inhibitors might be of potential clinical use in those cancers where these kinases identify a clinical subtype with a poorer prognosis, as is the case of VRK1 in breast cancer.

## Introduction

Most biological processes are regulated by reversible phosphorylation, and kinases play a central role in signal transmission. Kinases interconnect different signalling pathways in time and space, and confer flexibility to the regulation and coordination of multiple biological processes including cell division, apoptosis and survival among others. Furthermore, alteration in kinase function is a common underlying process to many pathological situations including cancer, inflammation, and neurodegeneration. The elucidation of the human kinome [1] has opened up new possibilities to characterize and develop strategies to manipulate these regulatory processes with therapeutic aims [2].

Kinase domains are very suitable for development of specific inhibitors [3], some of which have already been applied in cancer treatment, both for tyrosine kinases, such as PDGF/kit with imatinib in a variety of tumours, or to Ser-Thr kinases such as for B-Raf in melanomas [4]. Kinase domains in an inactive state are more structurally diverse than their activated form [2]. However, the main problem in development of specific inhibitors resides in the high conservation of the catalytic domain, which reduces the specificity of most inhibitors by targeting several kinases simultaneously, which makes them non specific [5], [6]. This cross-inhibition results in a significant promiscuity, which can be the cause of unexpected side effects in clinical use. The inhibition promiscuity of a kinase can be predicted based on the conservation of specific residues within the kinase fold [7].

The VRK kinase family received its name from vaccinia virus B1R, its unique kinase required for viral replication [8], [9]. The VRK family has a unique ortholog in *C. elegans* [10] and *D. Melanogaster* [11], but is composed of three proteins in mammals [1], a similar situation to the p53 family that has only one member in invertebrates and three members in mammals [12], which reflects the evolution of regulatory mechanisms as the organisms become more complex. These kinases in the human kinome belong to a unique and isolated subfamily with only three proteins VRK that very early, and near the kinases common trunk, diverged from the branch that much later led to casein kinase I family [1]. In addition, the VRK proteins have unique substitutions suggesting they might be pseudokinases [13]. VRK1 and VRK2 are two novel Ser-Thr kinases [14] that have a common catalytic domain with a fifty-three percent homology [13], [15], and play a role in cell division processes [16]–[18]. However, VRK1 [13], [19]–[21] and VRK2 [13], [22] have been demonstrated to be catalytically active; while VRK3, the most divergent of the three, is catalytically inactive [13]. Interestingly, the kinase activity of VRK1 and VRK2 proteins can be regulated by allosteric protein-protein interactions; they are both kinase-active when bound to RanGTP, and kinase-inactive when bound to RanGDP [23]. This indicates that these two kinases have two alternative conformations that can be allosterically regulated [23]. VRK1 is a nuclear kinase [13], [19], while VRK2 has two isoforms, a full-length protein of 508 aminoacids (known as VRK2 and VRK2A) [13], which is anchored to cytosolic organelle membranes, such as endoplasmic reticulum and mitochondria by its C-terminal hydrophobic anchoring region [22]; and VRK2B, with 397 aminoacids lacking the C-terminal region and detected both in cytosol and nucleus, perhaps functionally replacing in some aspects VRK1 and detected only in some cellular types, like adenocarcinomas [22]. The conservation in catalytic domain and different subcellular location indicate that substrate utilization, and perhaps specificity, might determine signal compartmentalization and substrate use. The regulation of kinases in time and space is likely to be an area of intense research in the future [24]. VRK1 is expressed at high levels in tumours with p53 mutations, such as in lung cancer [25] and identifies a subgroup of breast cancer with a poorer prognosis [26], [27]. VRK1 is the best characterized protein of the VRK family regarding its substrates, that include phosphorylation of p53 in T18 [16], [19], c-Jun in S63 and S73 [20], ATF2 in Ser62 and T73 [21], CREB1 in S133 [28] and histone H3 in T3 and S10 [23], [29], this latter modification regulates methylation and affects chromatin structure. Also, VRK1 functions as a coordinator of several processes required for cell division [18], identifies a bad prognosis signature in breast cancer [26], and specific expression patterns in human tissues, normal and malignant [30]. Kinase inhibitor screenings have not yet identified any inhibitor for the VRK family [31], consistent with its low promiscuity index [7]. Kinases can be discriminated using a small panel of thirty-eight inhibitors and three hundred and seventeen kinases as targets, including both tyrosine and serine-threonine kinases [31].

The atypical structure of VRK proteins determined by specific aminoacid substitutions [15] makes them suitable targets for development of specific inhibitors with reduced kinase promiscuity [7]. Therefore, in this work we have aimed to determine if catalytically active VRK1 and VRK2 proteins have similar or different sensitivity to current kinase inhibitors with the aim to obtain the starting point for future development of kinase specific inhibitors with limited or no cross-inhibition.

## Results

### Effect of kinase inhibitors on VRK1 and VRK2 kinase activity

**Figure 1:**
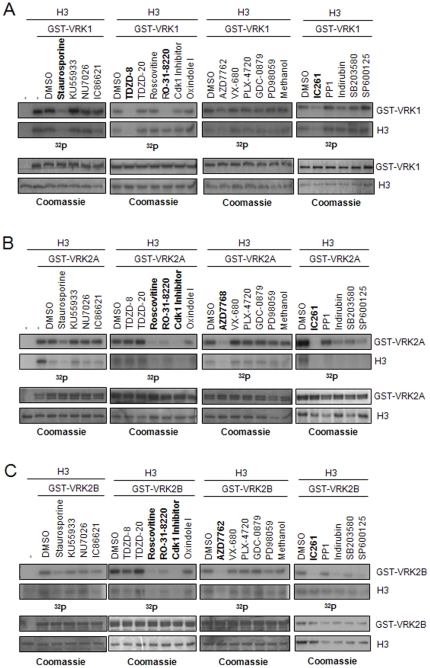
Effect of twenty different inhibitors on the kinase activity of human VRK1 (A), VRK2A (B) and VRK2B (C). The inhibition was assayed using both autophosphorylation (top), and phosphorylation of histone H3 (bottom), as targets in the kinase assay. All inhibitors were used at 100 µM. In bold are marked those inhibitors that appear to be somewhat more effective in the inhibition of each kinase. The top gels show the in vitro kinase assay with ATP (5 µM) and [γ-^32^P]-ATP (5 µCi), incubated for 30 minutes at 30°C. The bottom gels show the protein levels detected by Coomassie blue staining. The assays contain 2 µg of the indicated kinase and 1 µg of recombinant H3. TDZD-8 and TDZD-20 are non-competitive inhibitors. All the other compounds are competitive inhibitors.

Despite the similarity in the known in vitro substrates of VRK proteins, there are some differences in the primary aminoacid sequence of these kinases, suggesting that a possible way to functionally discriminate between VRK1 and VRK2 is by their sensitivity to kinase inhibitors. The VRK2 (VRK2A) crystal structure indicates that it initially has an active conformation, which is based on the structure of its kinase domain with its two lobes presenting a closed conformation, and an activation loop with a structure that is compatible with kinase activity [15], and has autophosphorylation activity [13], [22]. VRK1, in addition to its autophosphorylation [19], also phosphorylates histone H3 in Thr3 and Ser10 [23], [29]. As an initial approach, the effect of twenty inhibitors was determined at 100 µM (Fig. 1) and 500 µM (not shown) in order to identify which ones have some inhibitory effect on VRK1 or VRK2 kinase activity in the presence of 5 µM ATP, which permits a higher sensitivity to inhibitors [6], and it is a good initial screening, since those inhibitors which are effective in the micromolar range are highly unlikely to be of any use in vivo, since the intracellular ATP concentration is three orders of magnitude higher. Among these inhibitors, non-competitive and competitive, were included two that were detected to bind VRK1 and VRK2 proteins and identified by their induction of a thermal shift, such as oxindole I and Cdk1 inhibitor [15]. Their inhibitory effects were tested using an in vitro kinase assay based on autophosphorylation and histone H3 phosphorylation as substrate. Most of these inhibitors have little or no effect, but some differences were noticeable at these high concentrations of inhibitors. VRK1 was more sensitive to TDZD-8 (Fig. 1A) and VRK2 was more sensitive to roscovitine and Cdk1 inhibitor (Fig. 1B). The two kinases were somewhat sensitive to staurosporine, RO 31–8220, AZD7762 and IC261. Other inhibitors, such as TDZD-20 and oxindole I, were not able to inhibit either VRK1 or VRK2A. TDZD-8 and TDZD-20 are non competitive inhibitors. The inhibitor profile of VRK2B is similar to that of VRK2A (Fig. 1C) and this is consistent with the complete sequence identity of their common catalytic sites [22]. The summary of their IC_50_ values in the presence of 5 µM ATP is shown in Table 1.

**Table 1:** VRK1 and VRK2A sensitivity and IC50 values of serine-threonine kinase inhibitors.

	IC_50_ (µM)			
	GST-VRK1		GST-VRK2A	
	Auto- phosphorylation	H3 phosphorylation	Auto-phosphorylation	H3 phosphorylation
**Roscovitine**	No inhibition		25.7±1.23	55.34±1.6
**RO 31–8220**	11.11±1.16	34.39±1.05	31.77±1.15	33.63±1.04
**Cdk1 Inhibitor**	No inhibition		11.2±1.33	10.77±1.29
**Oxindole I**	No inhibition		No inhibition	
**Staurosporine**	15.27±1.10	36.87±1.09	No inhibition	
**KU55933**	No inhibition		No inhibition	
**NU7026**	No inhibition		No inhibition	
**IC86621**	No inhibition		No inhibition	
**AZD7768**	No inhibition		39.98±1.07	24.38±1.07
**VX-680**	No inhibition		No inhibition	
**PLX-4720**	No inhibition		No inhibition	
**GDC-0879**	No inhibition		No inhibition	
**PD98059**	No inhibition		No inhibition	
**IC261**	121.39±1.05	55.93±1.04	11.09±1.10	14.71±1.13
**PP1**	No inhibition		No inhibition	
**Indirubin**	No inhibition		50.66±1.23	57.23±1.17
**SB203580**	No inhibition		No inhibition	
**SP600125**	No inhibition		27.59±1.09	11.13±1.21
**TDZD-8**	Inhibition at 7.5 µM		No inhibition	
**TDZD-20**	No inhibition		No inhibition	

The IC50 values were calculated in the presence of 5 µM ATP. The individual points are the average from three experiments. The linear regression and the standard deviation were calculated with the SPSS program for VRK1 (Fig. S3) and VRK2A (Fig. S4). All values are µM. No inhibition is lack of effect at 100 µM.

**Figure 2:**
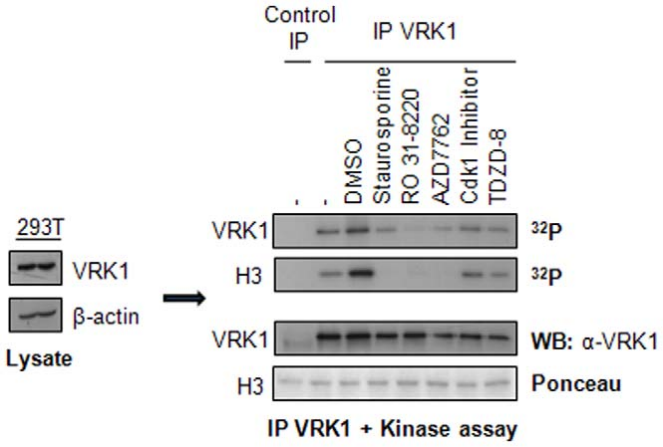
Inhibition of human VRK1 expressed in 293T cells. The endogenous VRK1 protein, from HEK 293T cells, was immunoprecipitated and used for an in vitro kinase assay. The relative inhibition was determined at a concentration of 75 µM for those inhibitors previously identified by testing them in bacterially expressed VRK1. Endogenous VRK1 protein was immunoprecipitated with 1F6 mAb, anti-VRK1, as control an anti-HA mAb was used. The VRK1 immunoprecipitate was incubated in the presence of 75 µM inhibitor, 5 µM ATP and 5 µCi of [γ^32^P]ATP.

The sensitivity of endogenous VRK1 to the inhibitors identified in kinase assays with bacterially expressed proteins was also determined. Endogenous VRK1 protein from 293T cell lysate was immunoprecipitated and used for kinase assays. The endogenous protein was sensitive to the same inhibitors as the purified protein (Fig. 2).

### VRK2 is more sensitive than VRK1 to CDK inhibitors

**Figure 3:**
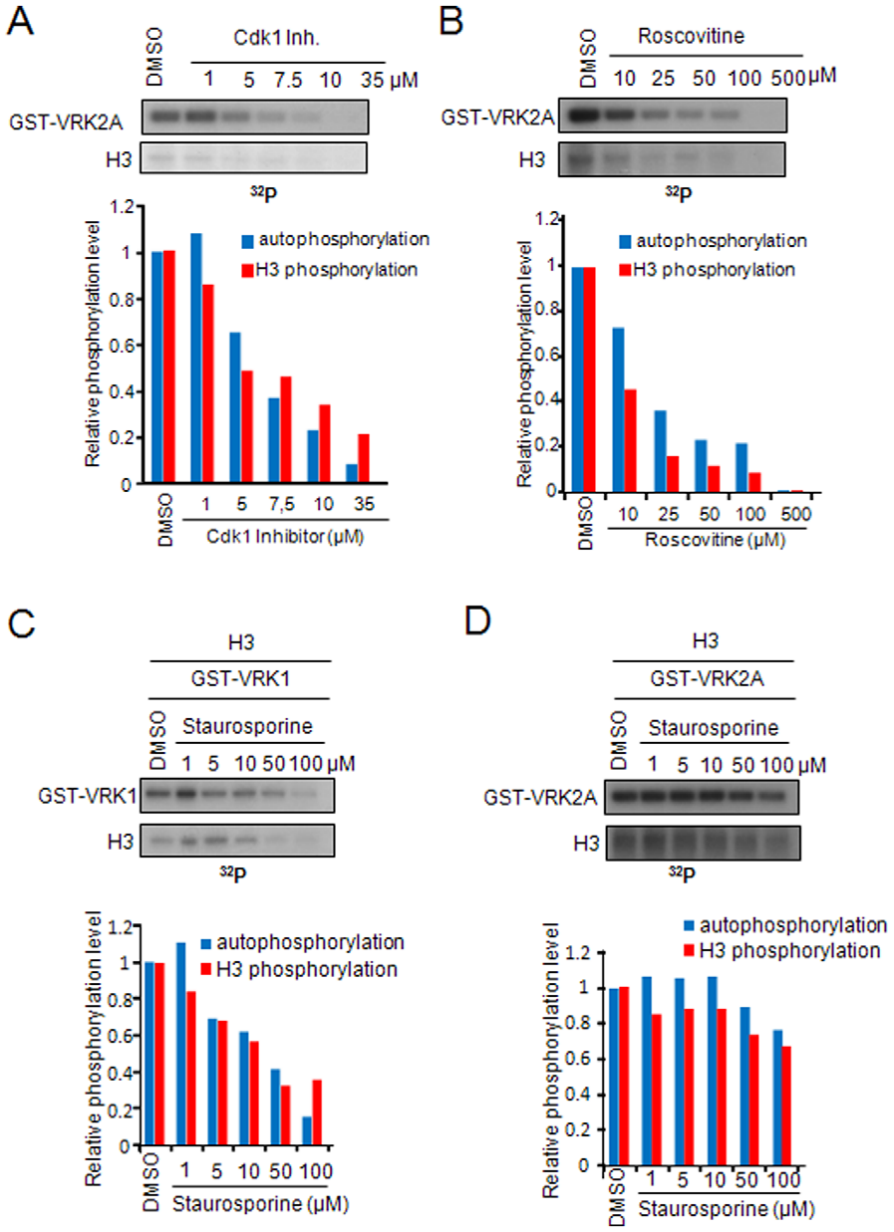
Differential effect of CDK inhibitors on VRK1 and VRK2 and discrimination between VRK1 and VRK2 by staurosporine. **A**. Inhibition of VRK2 by Cdk1 inhibitor. Quantification of the inhibition achieved on autophosphorylation and histone H3 phosphorylation is shown in the graph below. Quantification was performed in the linear response range. **B**. Inhibition of VRK2A by roscovitine, an inhibitor in phase II clinical trials. Quantification of the inhibition achieved on autophosphorylation and histone H3 phosphorylation is shown in the graph below. **C**. Inhibition of VRK1 activity by staurosporine. At the bottom the quantification in the linear response range is shown. **D**. Lack of effect of staurosporine on VRK2A activity. At the bottom the quantification in the linear response range is shown.

Next we proceeded to analyze in more detail the differential effect of inhibitors targeting CDK proteins such as Cdk1 Inhibitor, roscovitine and indirubin-3′-monoxime. Indirubin-3′-monoxime had little effect at the high concentration of 100 µM and was not studied any further (Fig. 1). VRK2 was more sensitive (Fig. 3A) to Cdk1 inhibitor than VRK1 (not shown), and the kinase activity of VRK2A was inhibited by fifty percent at 4 µM, determined in the presence of low ATP, which is similar to that on Cdk1/cyclinB (5.8 µM) [32]. It is important to note that the effect on both autophosphorylation and phosphorylation of H3 follow a similar pattern as shown in the graphs (Fig. 3A). Roscovitine (also known as CYC202 or Seliciclib), a pan-CDK inhibitor [33] currently in phase II clinical trials for breast and lung carcinomas [31], inhibited the activity of VRK2 (Fig. 3B) by fifty percent at approximately 25 µM, which is higher than the one reported for inhibition of CDK1/cyclinB, and CDK2/cyclinA (0.7 µM) [34]. VRK1 is less sensitive to roscovitine and was not inhibited at much higher concentrations, although at 250 µM there was some noticeable effect (Fig. 3D).

Also, a pan aurora inhibitor, VX-680 [33], in use in clinical trials [35] was tested without noticeable inhibitory effect on any VRK protein (Fig. 1).

### Effect of PKC inhibitors, RO 31-8220 and staurosporine, on VRK1 and VRK2 activity

Many inhibitors for PKC proteins have been reported. Among them are RO 31–8220 and staurosporine, which have been mainly tested on protein kinase C and can induce apoptosis [36], inhibit insulin secretion [37] and block PDGF response among many other effects that can require PKC [38]. The effect of RO 31–8220 was tested in kinase assays of VRK1 and VRK2A. For both kinases fifty percent inhibition was similar, between 11 to 34 µM on H3 phosphorylation or autophosphorylation activity, which is also much higher than the 5–27 nM reported for PKC isoforms [39],[40], although this inhibitor is known to inhibit multiple kinases such as MSK1, S6K1 and RSK [41], [42]. The effect on both autophosphorylation and phosphorylation of H3 followed a similar pattern (not shown).

In a wide screening, staurosporine appeared to be a potential inhibitor, although not very efficient, of VRK proteins [31]. VRK1 is more sensitive and fifty percent inhibition was achieved at 15 µM of staurosporine (Fig. 3C), which is much higher than the IC50 of 3 nM for PKC [43]. VRK2A was not inhibited by staurosporine (Fig. 3D). Therefore, staurosporine can discriminate between VRK1 and VRK2, which is an unexpected observation since staurosporine is one of the less specific inhibitors known [31].

### Effect of inhibitors targeting DNA damage response kinases: VRK2 is more sensitive than VRK1 to AZD7762

Cellular responses to DNA damage implicate many different kinases that might be suitable targets for pharmacological development, since they would sensitize cells to other chemotherapeutic drugs. Several inhibitors targeting ATM (KU 55933), DNA-PK (NU7026, IC86621), and CHK1/2 (AZD7762) were tested for their effect on VRK1 and VRK2A activity. Only AZD7762, an inhibitor targeting CHK1 and CHK2, two serine-threonine kinases involved in DNA damage responses [44], which is currently used in clinical trials [45], had some effect on VRK activity. Fifty percent inhibition of both VRK2A autophosphorylation and H3 phosphorylation was at 30 µM (not shown). VRK1 was less sensitive than VRK2A, and some inhibition was detectable at 100 µM (Fig. S1). The other inhibitors, KU 55933, NU7026 and IC86621 had no noticeable effect on VRK1 or VRK2A kinase activity (Fig. 1).

### Effect of casein kinase and MAPK inhibitors

VRK proteins are the closest group of kinases to casein kinase I family, from which they diverged very early [1]. IC261 is an inhibitor that targets several kinases such as CK1 [46]–[48]. Despite the closeness between the two VRK proteins, IC261 was more effective inhibiting VRK2A than VRK1, and VRK2A activity reached fifty percent inhibition at 10 µM (not shown).

Several inhibitors targeting p38 (SB203580), MEK1 (PD98059), B-Raf (PLX-4720 and GDC-0879) and JNK (SP600125) were tested. None of them was able to induce a significant inhibition of VRK1 or VRK2 activities at 100 µM (Fig. 1A,B). PP1, an inhibitor that targets several kinases such as Src, Lck and CK1δ [33], had no effect on VRK1 or VRK2 activities at 100 µM (Fig. 1A,B).

### Non-competitive inhibitors: VRK1 is more sensitive than VRK2 to TDZD-8

Heterocyclic thiadiazolidinones, TDZD-8 and TDZD-20, are two non-competitive inhibitors that were developed to inhibit GSK3β [49], and in clinical trials for treatment of Alzheimer's disease [50]. VRK1 was insensitive to this inhibitor, but in a very short concentration range its effect changed and VRK1 activity was fully inhibited. There was no significant inhibition of VRK1 activity at 5 µM, but it was almost completely inhibited at 7.5 µM, both in autophosphorylation or H3 phosphorylation (Fig. S2A). The related TDZD-20 inhibitor had no effect at similar concentrations (Fig. S2B). VRK2A was insensitive to TDZD-8 at 500 µM (not shown) and it was also insensitive to TDZD-20 at 100 µM (Fig. 1B).

### Inhibition of vaccinia virus B1R kinase

**Figure 4:**
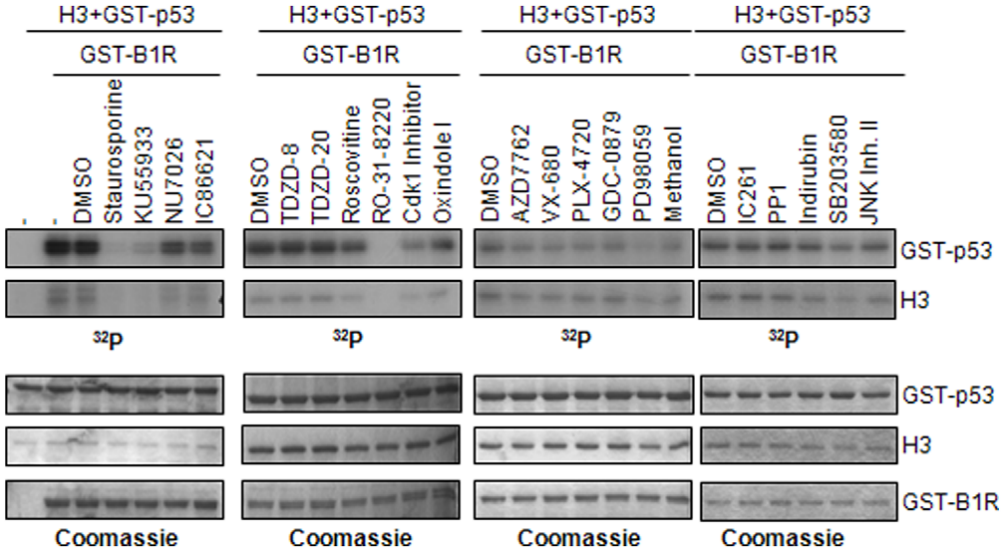
Inhibition of vaccinia virus B1R kinase. Phosphorylation assays were performed using 1 µg of purified GST-p53 or Histone H3 as substrates. Quantification was performed in the linear response range.

Vaccinia virus, and related poxviruses, has a unique kinase in their genome that is required for viral DNA replication [8], [9]. This kinase, B1R, gave the name to mammalian VRK proteins, but their homology is reduced to forty percent [13], and it presents differences in its phosphorylation activity compared to the human VRK proteins. B1R has a reduced autophosphorylation, and phosphorylates p53 in multiple residues [51], whereas VRK1 [16], [19] and VRK2 [22] phosphorylate p53 in a unique residue, and they also have a strong autophosphorylation activity. Therefore, it was tested the sensitivity of B1R to the panel of twenty kinase inhibitors in a kinase assay using p53 and histone H3 as substrates (Fig. 4) 5 in the presence of ATP at 5 µM. B1R was sensitive to staurosporine, KU55933 and RO 31–8220. This result has some overlap, but is not identical, to VRK1 or VRK2 inhibition patterns.

## Discussion

One of the main implications of VRK proteins is their potential utilization for developing specific inhibitors that may be used in oncologic treatments. But a main problem with current inhibitors is that they usually affect several related kinases simultaneously, although there might be some differences in affinity [5], [6], [33]. In practice, this means that the clinical use of inhibitors affecting several kinases might present a significant risk of uncontrolled side effects. An alternative approach to identify kinases for specific targeting is the use of kinase specific siRNA [52]. VRK proteins were not identified in an extensive kinase siRNA screening [53], probably because the effect was studied at forty-eight hours, which is not suitable for very stable proteins with half-life of four to six days such as VRK1 [54]. However, kinases knockdown has a limitation in case of very stable proteins, as VRKs, since in RNA interference experiments the observation time allows the reduction in RNA, but not in the protein level [53]. The knockdown of VRK1 and VRK2 has already provided indication of processes that might be selectively affected by their specific inhibition. Knockdown of VRK1 results in a block in cell cycle progression before the restriction point in G1 [16], [54], thus it can be used in pathologies where proliferation is part of its pathogenesis. In the case of VRK2, its knockdown affects signalling by MAPK, since VRK2 modulates signal transmission by direct interaction with scaffold proteins, such as JIP1 affecting the response to hypoxia [55] or cytokines [56], and KSR1 affecting oncogene signalling [57].

Based on their structural differences, VRK1 and VRK2 kinases are predicted to be proteins with a very low promiscuity index and be insensitive to current kinase inhibitors [5], [7], [31]. The pattern of VRK inhibitors detected in this work confirms this prediction and presents two main characteristics. First of all, human VRK1 and VRK2, as well as vaccinia B1R, are in general very insensitive to the panel of inhibitors tested in the current study that target a large variety of human kinases with an IC50 in the nanomolar range in most cases. Most of them have little, if any, effect on VRK kinases even at a high concentration, which makes them unsuitable for in vivo use. The second characteristic is that the inhibition detected for some compounds does not bear any relation to a particular subtype of kinases.

Among the poor inhibitors identified, there is a clear differential pattern between VRK1 and VRK2. VRK1 is more sensitive to staurosporine and RO 31–8220, two inhibitors of PKC; while VRK2 is more sensitive to Cdk1 inhibitor and roscovitine, two Cdk1 (cdc2) inhibitors. Interestingly, Cdk1 inhibitor has been shown to equally interact with both kinases [15], but only VRK2 activity was inhibited. For all inhibitors, their sensitivity is reduced by three orders of magnitude when compared with their preferentially targeted kinases. Another inhibitor for which VRK proteins show some sensitivity is AZD7762 that targets CHK1 and CHK2 with much higher affinity. Although VRK2, and less efficiently VRK1, are inhibited by AZD7762, the IC50 is more than five orders of magnitude higher than that required for CHK1 and CHK2 inhibition [44], [58]. Thus, IC261 inhibits CK1 at 6 micromolar, similar to the inhibition of VRK2 [5], [33], but has no effect on VRK1 activity. In addition, VRK1, but not VRK2, is sensitive to a non-competitive inhibitor TDZD-8, which targets GSK3. Neither VRK1 nor VRK2 respond to current inhibitors of B-Raf (wild-type or mutant V600E), ATM, DNA-PK, MEK1 and aurora kinases. The observation that even the best inhibitors only have some effect at low micromolar concentrations, when they are assayed in the presence of 5 µM ATP, indicates that both substrate and inhibitor have to be at similar concentrations in order to detected an inhibitory effect, and this means that in vivo the inhibitor is not likely to function since intracellular ATP concentration is three orders of magnitude higher.

These data suggest that a comparative analysis of VRK2 structure with that of those inhibitors to which they are somewhat sensitive might provide enough structural clues that can be used to start modelling VRK1 and VRK2 specific inhibitors with a reduced promiscuity. The differences detected in the kinase domain of VRK proteins indicate that they might be very suitable for designing specific inhibitors, because the likelihood of cross-inhibition of other kinases is very low, as suggested by the promiscuity score in which VRK1 and VRK2 are the kinases with the likelihood of having the most specific inhibitors [7]. This prediction was also confirmed in a different experimental approach based on the determination on the kinase specificity of current inhibitors [31]. VRK1 has been identified as a drugable kinase in rhabdomyosarcoma [59] and breast cancer [60].

The pattern of VRK1 and VRK2 inhibition suggests that they might be structurally closer to cdk1 than any other kinases, but even so, they maintain large differences. However, the high concentrations needed to achieve some inhibition means that none of the inhibitors tested can be used to inhibit VRK proteins in cell based assays, since they will also affect several other kinases.

Kinase activation implies a conformational change involving the activation loop that has a DFG motif in an out (inactive) or in (active) state [61], [62]. These alternative conformations might affect the kinase response to inhibitors. In the DFG out or inactive state, the kinase might bind and prevent the activating conformational change, rather than displacing ATP in case of competitive inhibitors. Thus, depending on the conformation the effect may vary. On the other hand, in the active state, competitive inhibitors will displace the nucleotide. In vivo the situation is likely to be a mixture of different situations. VRK1 inhibition by TDZD-8, a non competitive inhibitor of GSK3β [49], might be a particular case. The TDZD-8 effect on VRK1 activity seems to be an all or none effect at a specific concentration. This might reflect the switch between two alternative VRK1 conformations when the inhibitor reaches a critical threshold concentration. It would be interesting to know if TDZD-8 is acting by maintaining a loop out conformation for its activation loop [61] that has some peculiarities [15], [62].

The identification and validation of specific inhibitors for human VRK proteins and vaccinia B1R have the potential of clinical applications. In this context, development of specific inhibitors for VRK1 and VRK2 is a real possibility because they are likely to be highly specific. Since these kinases have been implicated in response to growth factors and in DNA damage response, their inhibitors can make cells more sensitive to current chemotherapeutic drugs or irradiation, reducing the toxicity associated with them, since kinase inhibitors have shown to be well tolerated by patients. Use of kinase inhibitors for treatment of acute infection by poxviruses, such as smallpox, might be an alternative therapy for acute viral infection by reducing viral replication. The development of such specific inhibitors is a real possibility that needs to be pursued once the structure of these proteins and lead compounds become available.

## Materials and Methods

### Plasmids and expression of proteins

Human VRK1 was expressed from plasmid pGEX4T-VRK1 [19] and purified using Glutathion-Sepharose (GE Healthcare). VRK2A and VRK2B proteins were expressed from plasmids pGEX4T-VRK2A and pGEX4T-VRK2B respectively in BL21 *E. coli* strain [22]. Vaccinia virus B1R was expressed from plasmid pGEX-B1R [51]. The GST-p53 has been described previously [19], [51]. GST fusion proteins were eluted from the corresponding resin with reduced glutathione [22]. Protein purification was checked in a 10% PAGE [63]. Endogenous VRK1 protein from 293T cells was immunoprecipitated with an anti-VRK1 monoclonal antibody (1F6) [63], and the immunoprecipitate was used for an in vitro kinase assay.

### Reagents

All reagents were of analytical grade from Sigma. The nucleotide [^32^P-γ] ATP was from PerkinElmer/NEN. Recombinant histone H3 was from Upstate Biotechnology-Millipore (Lake Placid, NY).

### *In vitro* kinase assay

Kinase assays were performed using both purified proteins and histone H3, or immunoprecipitated candidate proteins. VRK kinase activity was determined by assaying protein phosphorylation in a final volume of 30 µL containing kinase buffer (20 mM Tris-HCl pH 7.5, 5 mM MgCl_2_, 0.5 mM DTT and 150 mM KCl), 5 µM ATP and 5 µCi of [γ^32^P]ATP with 2 µg of GST-VRK1, GST-VRK2A or GST-VRK2B protein and the indicated concentrations of kinase inhibitors. In this work we used bacterially expressed VRK1, as well as immunoprecipitated endogenous VRK1, and 1 µg of recombinant histone H3 was used as a substrate. The kinase, substrate H3 and inhibitor were pre-incubated for 10 min at 30°C before adding ATP. In the case of vaccinia B1R protein that has a low autophosphorylation activity, 1 µg of GST-p53 was used as substrate. Then, the reactions were performed at 30°C for 30 min in a Thermomixer (Eppendorf) and stopped by boiling in Laemmli buffer. Reactions and quantifications were performed in their linear response range. The proteins in the assay were analyzed by electrophoresis in 12.5% SDS-polyacrilamide gels. The gels were stained with Coomassie Blue or proteins were transferred to PVDF membrane and the incorporated activity was measured. The SPSS program v.19 (Inc. IBM Company) was used for linear regression analysis and calculation of IC50 values.

### Kinase inhibitors

Roscovitine; Cdk1 Inhibitor; Oxindole I; IC261/SU 5607, an inhibitor of CK1; PP1, an inhibitor of LCK and FYN; PD 98059, a selective, reversible inhibitor of MEK; and SP600125 inhibitor of JNK, were from Calbiochem-Merck (Darmstadt, Germany). NU7026, an inhibitor of DNA-PK in a ATP-competitive manner; IC86621, a DNA-PK catalytic subunit inhibitor; SB 203580, inhibitor of p38; Indirubin-3′-monoxime, an inhibitor of CDK; Staurosporine, a potent inhibitor of PKC; and RO 31–8220 were from Sigma-Aldrich (St. Louis, MO). KU 55933 a selective and competitive ATM kinase inhibitor that functions as a radio- and chemosensitizer for cancer treatment, was from Tocris Bioscience (Bristol, UK). VX-680, an Aurora kinases inhibitor; AZD7762, inhibitor of CHK1/2; PLX-4720 and GDC-0879, B-raf1 inhibitors were from Selleck Chemicals (Houston, TX). Non competitive inhibitors: TDZD-8 (GSK-3β Inhibitor I), and TDZD-20 (GSK-3 Inhibitor XVII) were from Calbiochem-Merck (Darmstadt, Germany). Inhibitors are summarized in Table S1.

## Supporting Information

Figure S1 **Effect of AZD7762, a CHK1/2 inhibitor on VRK1 (A) and VRK2 (B).** At the bottom the quantification in the linear response range is shown. VRK2A is more sensitive than VRK1 to this inhibitor independently of the assay type. AZD7762 is currently in phase II clinical trials.
(TIF)

Figure S2 **Effect of TDZD-8 and TDZD-20 non-competitive inhibitors on VRK1 and VRK2.** A. Effect of TDZD-8 on VRK1 in autophosphorylation and H3 phosphorylation assays. At the bottom the quantification of the blots is shown. B. Effect of TDZD-20 on VRK1 autophosphorylation and H3 phosphorylation. C. Effect of TDZD-8 on VRK2A autophosphorylation and H3 phosphorylation.
(TIF)

Figure S3 **Determination of IC50 values for several inhibitors in autophosphorylation and histone H3 transphosphorylation assays of VRK1.** The values from three experiments using inhibitors to which VRK1 is sensitive were used for calculation of the IC50 value. Linear regression analysis was performed and the R^2^ value calculated using the SPSS program.
(TIF)

Figure S4 **Determination of IC50 values for several inhibitors in autophosphorylation and histone H3 transphosphorylation assays of VRK2A.** The values from three experiments using inhibitors to which VRK2A is sensitive were used for calculation of the IC50 value. Linear regression analysis was performed and the R^2^ value calculated using the SPSS program.
(TIF)

Table S1 **Inhibitors of serine-threonine kinases**
(DOC)
