# Supplementary figures and images for: Differential Inhibitor Sensitivity between Human Kinases VRK1 and VRK2
Source: PLoS One. 2011 Aug 4;6(8):e23235. doi: 10.1371/journal.pone.0023235 (PMC3150407; doi:10.1371/journal.pone.0023235)

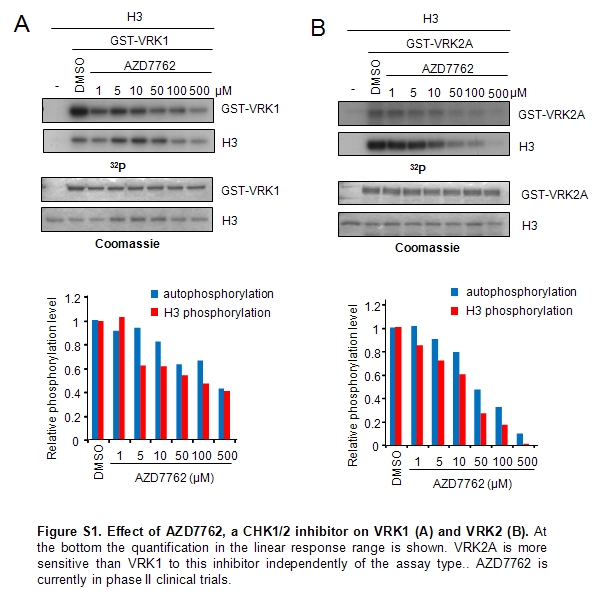

Supplement: Figure S1 — Effect of AZD7762, a CHK1/2 inhibitor on VRK1 (A) and VRK2 (B). At the bottom the quantification in the linear response range is shown. VRK2A is more sensitive than VRK1 to this inhibitor independently of the assay type. AZD7762 is currently in phase II clinical trials. (TIF) [file pone.0023235.s001.tif]

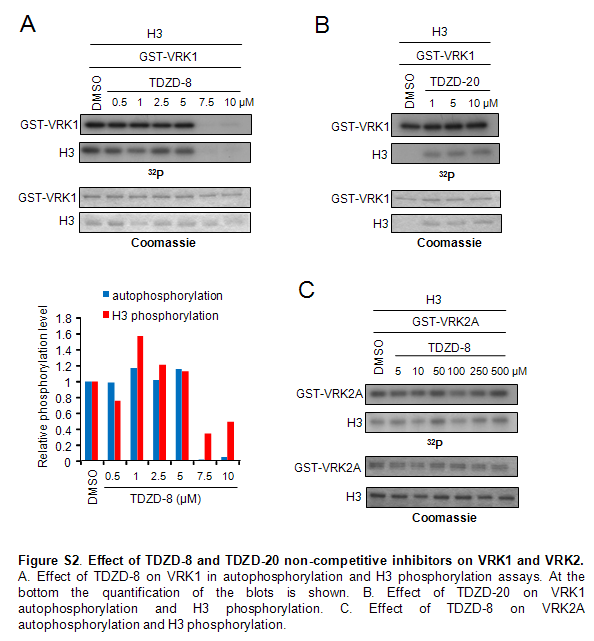

Supplement: Figure S2 — Effect of TDZD-8 and TDZD-20 non-competitive inhibitors on VRK1 and VRK2. A. Effect of TDZD-8 on VRK1 in autophosphorylation and H3 phosphorylation assays. At the bottom the quantification of the blots is shown. B. Effect of TDZD-20 on VRK1 autophosphorylation and H3 phosphorylation. C. Effect of TDZD-8 on VRK2A autophosphorylation and H3 phosphorylation. (TIF) [file pone.0023235.s002.tif]

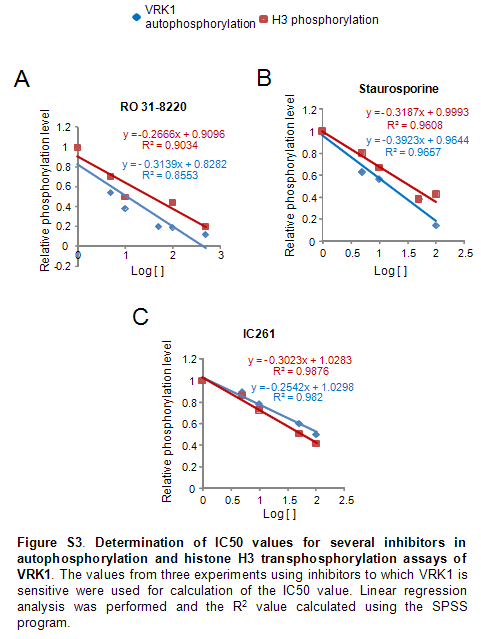

Supplement: Figure S3 — Determination of IC50 values for several inhibitors in autophosphorylation and histone H3 transphosphorylation assays of VRK1. The values from three experiments using inhibitors to which VRK1 is sensitive were used for calculation of the IC50 value. Linear regression analysis was performed and the R2 value calculated using the SPSS program. (TIF) [file pone.0023235.s003.tif]

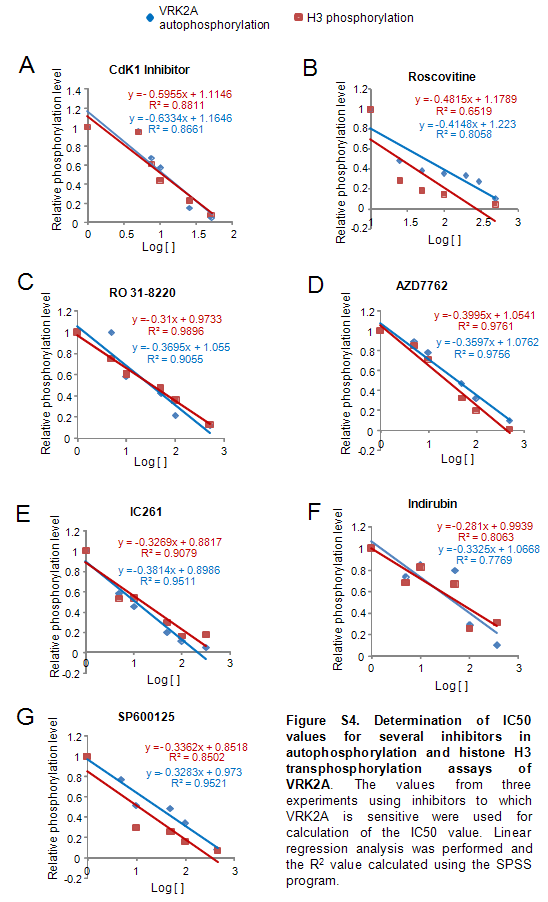

Supplement: Figure S4 — Determination of IC50 values for several inhibitors in autophosphorylation and histone H3 transphosphorylation assays of VRK2A. The values from three experiments using inhibitors to which VRK2A is sensitive were used for calculation of the IC50 value. Linear regression analysis was performed and the R2 value calculated using the SPSS program. (TIF) [file pone.0023235.s004.tif]
