# Supplementary material for: Differential Inhibitor Sensitivity between Human Kinases VRK1 and VRK2
Source: PLoS One. 2011 Aug 4;6(8):e23235. doi: 10.1371/journal.pone.0023235 (PMC3150407; doi:10.1371/journal.pone.0023235)
Supplement: Table S1 — Inhibitors of serine-threonine kinases (DOC) [file pone.0023235.s005.doc]

TABLE S1 : **Serine-threonine inhibitors tested on VRK proteins**

| **Inhibitor** | **Kinase target (IC50)** | **Supplier** |
| --- | --- | --- |
| **VX-680**: N-[4-[4-(4-Methylpiperazin-1-yl)-6-(3-methyl-1H-pyrazol-5-ylamino)pyrimidin-2-sulfanyl]phenyl]- cyclopropanecarboxamide | Aurora A kinase (0,6 nM)  Aurora B kinase (18,4 nM)  Aurora C kinase (6 nM) | Selleck Chemicals |
| **TDZD-8**: 4-benzil-2methyl-1,2,4-thiodiazolidine-3,5-dione | GSK-3β (2 µM) | Calbiochem |
| **TDZD-20**: 5-Benzylamino-3-oxo-2,3-dyhidro-1,2-4-thiadiazole | GSK-3β (6,5 µM) | Calbiocehm |
| **Cdk1 Inhibitor**: 3-(2-Chloro-3-indolylmethylene)-1,3-dihydroindol-2-one | Cdk1 (5,8 µM) | Calbiochem |
| **Roscovitine**: 2-(R)-(1-Ethyl-2-hydroxyethylamino)-6-benzylamino-9-isopropylpurine | Cdk1 (650 nM) | Calbiochem |
| **Indirubin-3’-monoxime** | CDK (50-100 nM), GSK3β (5-50 nM) | SIGMA |
| **Staurosporine**: (*9S,10R,11R,13R*)-2,3,10,11,12,13-Hexahydro-10-methoxy-9-methyl-11-(methylamino)-9,13-epoxy-1*H*,9*H*-diindolo[1,2,3-gh:3',2',1'-lm]pyrrolo[3,4-j][1,7]benzodiazonin-1-one | PKC (3 nM) | SIGMA |
| **Ro 31-8220**: 2-(1-[3-(Amidinothio)propyl]-1H-indol-3-yl)-3-methanosulfonate salt | PKC isoforms (5-27 nM) | SIGMA |
| **Oxindole I**: 3-(1H-Pyrrol-2-ylmethylene)-1,3-dihydroindol-2-one | Flk-1 (390 nM) | Calbiochem |
| **KU 55933**: 2-(4-Morpholinyl)-6-(1-thianthrenyl)-4*H*-pyran-4-one | ATM (2,2 nM) | TOCRIS bioscience |
| **NU7026**: 2-(Morpholin-4-yl)-benzo[h]chomen-4-one | DNA-PK (230 nM) | SIGMA |
| **IC86621**: 1-(2-Hydroxy-4-morpholin-4-yl-phenyl)ethanone | DNA-PKcs (100 nM) | SIGMA |
| **AZD7762**: 5-(3-Fluorophenyl)-3-ureidothiophene-N-[(S)-piperidin-3-yl]-2-carboxamide | CHK1 (5 nM)  CHK2 (<10 nM) | Selleck Chemicals |
| **PD98059**: 2’-Amino-3’-methoxyflavone | Mek1 (4 µM) | MERCK |
| **IC261/SU 5607**: 3-[2,4,6-Trimethoxyphenyl)methylidenyl]-indolin-2-one | CK1, S6K, MSK1 (6 µM) | Calbiochem |
| **PP1**: 4-amino-5-(4-methylphenyl)-7-(t-butyl)pyrazolo-d-3,4-pyridine | LCK and FYN (5 µM), CK1δ (1 µM) | Calbiochem |
| **PLX-4720**: N-(3-(5-chloro-1H-pyrrolo[2,3-b] pyridine-3-carbonyl)-2,4-difluorophenyl)propane-1-sulfonamide | B-RafV600E (13 nM)  B-Raf (160 nM) | Selleck Chemicals |
| **GDC-0879**: 5-[1-(2-Hydroxy-ethyl)-3-pyridin-4-yl-1H-pyrazol-4-yl]-indan-1-one oxime | B-RafV600E (0,13 nM) | Selleck Chemicals |
| **SP600125**: Anthra[1,9-*cd*]pyrazol-6(2*H*)-one, 1,9-pyrazoloanthrone | JNK (40 nM) | Calbiochem |
| **SB 203580**: 4-(4-Fluorophenyl)-2-(4-methylsulfinylphenyl)-5-(4-pyridyl)-1H-imidazole | p38 (34 nM) | SIGMA |
